# Theoretical and Experimental Designs on Several Mechanical Properties of Cu–Al–Zn Shape Memory Alloys Used in the Processing Industry

**DOI:** 10.3390/ma16041441

**Published:** 2023-02-08

**Authors:** Constantin Plăcintă, Sergiu Stanciu, Mirela Panainte-Lehadus, Emilian Mosnegutu, Florin Nedeff, Valentin Nedeff, Claudia Tomozei, Tudor-Cristian Petrescu, Maricel Agop

**Affiliations:** 1 Faculty of Material Science and Engineering, “Gheorghe Asachi” Technical University of Iasi, 700050 Iasi, Romania; 2 Department of Environmental Engineering and Mechanical Engineering, Faculty of Engineering, Vasile Alecsandri University of Bacau, 157 Calea Marasesti, 600115 Bacau, Romania; 3 Gheorghe Ionescu Sisesti, Academy of Agricultural and Forestry Sciences Bucharest, 61 Marasti, 011464 Bucharest, Romania; 4 Faculty of Civil Engineering and Building Services, “Gheorghe Asachi” Technical University of Iasi, 700050 Iasi, Romania; 5 Department of Physics, “Gheorghe Asachi” Technical University of Iasi, 700050 Iasi, Romania; 6 Romanian Scientists Academy, 54 Splaiul Independentei, 050094 Bucharest, Romania

**Keywords:** shape memory alloy, mechanical behavior, hysteresis, multifractal tunnel effect, multifractality, Scale Relativity Theory

## Abstract

By assimilating shape memory alloys with mathematical multifractal-type objects, a theoretical model based on Scale Relativity Theory in the form of The Multifractal Theory of Motion, in order to explain the mechanical behavior of such material, is proposed. The model is validated by analyzing the mechanical behavior of Cu–Al–Zn shape memory alloy with various chemical compositions. More precisely, the multifractal tunnel effect can “mime” the mechanical hysteresis of such a material, a situation in which a direct correspondence for several mechanical properties of Cu–Al–Zn is highlighted (the chemical composition can be correlated with the shapes of the curves controlled through the multifractality degree, while the areas delimited by the same curves can be correlated with the multifractal specific potential, as a measure of the mechanical memory degree).

## 1. Introduction

An important direction in the development of equipment used in the processing industry is the use of devices whose structurality and functionality are based on a category of materials, called shape memory alloys (SMAs) [1,2,3,4,5].

SMAs possess a collection of qualities that are markedly distinct, in contrast with usual metallic materials. A primary trait is the capacity to alter their geometric shape when exposed to temperature variations. In specific circumstances, this shape altering can be reversible, in such a manner that the material can ”memorize” two geometric shapes: a high-temperature shape and a low-temperature shape. These are also known as the “warm” shape and the “cold” shape, respectively. The mentioned transformations take place by virtue of an effect referred to as the shape memory effect (SME). Additionally, by means of SME, the material is capable of producing mechanical work when a shift from one shape to the other takes place [6].

Many types of SMAs are very expensive, due to their composition (which includes noble metals) and their complex manufacturing technologies. The most commonly encountered alloys currently are Ni–Ti, Cu–Al–Zn and Cu–Al–Ni, which are employed in a plethora of practical applications.

Shape memory alloys can be classified into the following groups according to the nature of the chemical elements found in their composition:

1. Alloys based on Ni–Ti [2,7,8,9];
2. Copper-based alloys (Cu–Al–Zn, Cu–Al–Ni) [10,11,12,13];
3. Ferrous alloys (Fe–Mn, Fe–Ni–C) [14,15,16,17,18,19];
4. Noble alloys (Au–Cd, Ag–Cd) [20,21,22];
5. ”Exotic” alloys (In–Te, In–Cd, V–Nb, etc) [23,24,25,26,27,28,29].

From all these, the most commonly employed are the ones based on Ni–Ti and Cu. Most methods for obtaining SMAs have three main stages: elaboration, primary thermal treatment and obtaining ”cold” and ”hot” shapes [30,31]. The elaboration processes are differentiated based on the type of alloy and, within the same type of alloy, on the desired properties.

Shape memory alloys have a multitude of applications, being found in a wide range of products (glasses frames, mobile phone antennas, super-elastic Ni–Ti powder to strengthen the strength of SnPdAg solder, SMA actuators for transmission fluid control, thermal switch, micro-electro-mechanical devices, sensors, anti-corrosion protective layers, etc.), but also in broad research areas (vibration and damping devices, biomedical, etc.) [1,2,3,4,5,32,33,34,35,36].

From such a perspective, the scope of the present work is to propose a mathematical model, based on Scale Relativity Theory in the form of The Multifractal Theory of Motion, to explain some mechanical behaviors of SMA systems. The model is validated by analyzing mechanical hysteresis-type behaviors of Cu–Al–Zn shape memory alloys, with various chemical compositions.

## 2. Theoretical Design

Given both the novelty and the complexity of the operational procedures that are involved in such mathematical models, it is necessary, in our opinion, to present some consequences of non-differentiability applied to SMA dynamics.

### 2.1. Non-Differentiability Applied to SMA Dynamics in the Form of the Multifractal Hydrodynamic Model: A Short Reminder

Let it be considered that any shape memory alloy (SMA), as a complex system, can be assimilated to a mathematical object of multifractal or fractal type. Then, the dynamics of such a material are explained by means of the Scale Relativity Theory, in the form of The Multifractal Theory of Motion. In such circumstances, the SMA structural unit (or entity) dynamics manifest on multifractal/fractal curves (i.e., continuous but non-differentiable curves). In the formalism of the Scale Relativity Theory, these dynamics can be portrayed by means of the scale covariance derivative [37,38,39]:

(1) $$\frac{\hat{d}}{dt}=\partial_{t}+{\hat{V}}^{l}\partial_{l}+\frac{1}{4}{\left(dt\right)}^{\left[\frac{2}{f\left(\alpha \right)}\right]-1}D^{lp}\partial_{l}\partial_{p},$$

where:

(2a) $${\hat{V}}^{l}=V_{D}^{l}-V_{F}^{l}$$

(2b) $$D^{lp}=d^{lp}-i{\hat{d}}^{lp}$$

(2c) $$d^{lp}=\lambda_{+}^{l}\lambda_{+}^{p}-\lambda_{-}^{l}\lambda_{-}^{p}$$

(2d) $${\hat{d}}^{lp}=\lambda_{+}^{l}\lambda_{+}^{p}+\lambda_{-}^{l}\lambda_{-}^{p}$$

In the above equations, the variables and parameters that describe the SMA dynamics have their meanings given and explained in [37,38,39], while various definitions for fractal dimensions, singularity spectrum f(α) of order α, etc. are found in [40,41].

In conditions where fractalization/multifractalization can be realized through Markov-type stochasticization, then the following relations become functional [37,38,39,42]:

(3) $$\lambda_{+}^{i}\lambda_{+}^{l}=\lambda_{-}^{i}\lambda_{-}^{l}=2\lambda \delta^{il}\;\;\;\;i,\;l=1,2,3$$

In Equation (3), the coefficient *λ* and the pseudo-tensor *δ*^*il*^ are specified in [38,39]. With these constraints, the scale covariant derivative Equation (1) takes the form:

(4) $$\frac{d}{dt}=\partial_{t}+{\hat{V}}^{l}\partial_{l}-i\lambda {\left(dt\right)}^{\left(\frac{2}{f\left(\alpha \right)}\right)-1}\partial_{l}\partial^{l}$$

In what follows, let it be accepted the motion principle given in [37,38,39]. Consequently, the motion equations of the SMA’s structural unit dynamics are:

(5) $$\frac{d{\hat{V}}^{i}}{dt}=\partial_{t}{\hat{V}}^{i}+{\hat{V}}^{l}\partial_{l}{\hat{V}}^{i}+\frac{1}{4}{\left(dt\right)}^{\left[\frac{2}{f\left(\alpha \right)}\right]-1}D^{lk}\partial_{l}\partial_{k}{\hat{V}}^{i}=0$$

From here, Equation (5) with the Equation (3) takes either the form of:

(6a) $$\frac{\hat{d}{\hat{V}}^{i}}{dt}=\partial_{t}{\hat{V}}^{i}+{\hat{V}}^{l}\partial_{l}{\hat{V}}^{i}-i\lambda {\left(dt\right)}^{\left[\frac{2}{f\left(\alpha \right)}\right]-1}\partial_{l}\partial^{l}{\hat{V}}^{i}=0$$

in the case of multifractal manifolds, or of:

(6b) $$\frac{\hat{d}{\hat{V}}^{i}}{dt}=\partial_{t}{\hat{V}}^{i}+{\hat{V}}^{l}\partial_{l}{\hat{V}}^{i}-i\lambda {\left(dt\right)}^{\left[\frac{2}{D_{F}}\right]-1}\partial_{l}\partial^{l}{\hat{V}}^{i}=0$$

in the case of a monofractal manifold.

- Therefore, according to [37,38,39,42], the existence of either the term $i\lambda {\left(dt\right)}^{\left(\frac{2}{f\left(\alpha \right)}\right)-1}$ from Equation (6a) or $i\lambda {\left(dt\right)}^{\left[\frac{2}{D_{F}}\right]-1}$ from Equation (6b) may correspond to a complex viscosity coefficient, the first being of a multifractal type and the second being only of a monofractal type. In such a context, it is possible to state that either the multifractal fluid or the fractal fluid, which describes the SMA dynamics of such a material, has a “memory”.

The separation of the SMA’s entities’ dynamics on scale resolutions (both the differentiable and the non-differentiable scale resolutions) Equation (5) takes the form:

(7a) $$\partial_{t}V_{D}^{i}+V_{D}^{l}\partial_{l}V_{D}^{i}-V_{F}^{l}\partial_{l}V_{F}^{i}+\frac{1}{4}{\left(dt\right)}^{\left[\frac{2}{f\left(\alpha \right)}\right]-1}D^{lk}\partial_{l}\partial_{k}V_{D}^{i}=0$$

(7b) $$\partial_{t}V_{F}^{i}+V_{F}^{l}\partial_{l}V_{D}^{i}+V_{D}^{l}\partial_{l}V_{F}^{i}-\frac{1}{4}{\left(dt\right)}^{\left[\frac{2}{f\left(\alpha \right)}\right]-1}D^{lk}\partial_{l}\partial_{k}V_{F}^{i}=0$$

while Equation (6a) takes the form:

(8a) $$\partial_{t}V_{D}^{i}+V_{D}^{l}\partial_{l}V_{D}^{i}-\left[V_{F}^{l}+\lambda {\left(dt\right)}^{\left[\frac{2}{f\left(\alpha \right)}\right]-1}\partial^{l}\right]\partial_{l}V_{F}^{i}=0$$

(8b) $$\partial_{t}V_{F}^{i}+V_{D}^{l}\partial_{l}V_{F}^{i}+\left[V_{F}^{l}+\lambda {\left(dt\right)}^{\left[\frac{2}{f\left(\alpha \right)}\right]-1}\partial^{l}\right]\partial_{l}V_{D}^{i}=0$$

An interesting case, with implications in SMA dynamics, is the one of irrotational dynamics. For such a situation, Equation (2a) becomes:

(9) $${\hat{V}}^{i}=-2i\lambda {\left(dt\right)}^{\left[\frac{2}{f\left(\alpha \right)}\right]-1}\partial^{i}\ln\Psi$$

where Ψ represents the states function. Further on, for (Madelung’s procedure):

(10) $$\Psi =\sqrt{\rho }e^{is}$$

where $\sqrt{\rho }$ is the amplitude and *s* is the phase, Equation (9) becomes:

(11) $${\hat{V}}^{i}=2\lambda {\left(dt\right)}^{\left[\frac{2}{f\left(\alpha \right)}\right]-1}\partial^{i}s-i\lambda {\left(dt\right)}^{\left[\frac{2}{f\left(\alpha \right)}\right]-1}\partial^{i}\ln\rho$$

or, moreover:

(12) $$V_{D}^{i}=2\lambda {\left(dt\right)}^{\left[\frac{2}{f\left(\alpha \right)}\right]-1}\partial^{i}s$$

and

(13) $$V_{F}^{i}=i\lambda {\left(dt\right)}^{\left[\frac{2}{f\left(\alpha \right)}\right]-1}\partial^{i}\ln\rho$$

By Equations (12) and (13) and employing the mathematical methodology from [38,42], Equations (8a) and (8b) reduce to the multifractal hydrodynamic equations:

(14) $$\partial_{t}V_{D}^{i}+V_{D}^{l}\partial_{l}V_{D}^{i}=-\partial^{i}Q$$

(15) $$\partial_{t}\rho +\partial_{l}\left(\rho V_{D}^{l}\right)=0$$

with *Q* being the multifractal specific potential:

(16) $$Q=-2\lambda^{2}{\left(dt\right)}^{\left[\frac{4}{f\left(\alpha \right)}\right]-2}\frac{\partial^{l}\partial_{l}\sqrt{\rho }}{\sqrt{\rho }}=-V_{F}^{i}V_{F}^{i}-\frac{1}{2}\lambda {\left(dt\right)}^{\left[\frac{2}{f\left(\alpha \right)}\right]-1}\partial_{l}V_{F}^{l}$$

Equation (14) defines the multifractal specific momentum conservation law of SMA dynamics. Equation (15) defines the multifractal state density conservation law for the same dynamics. The quantity *Q* generates the multifractal specific force:

(17) $$F^{i}=-\partial^{i}Q=-2\lambda^{2}{\left(dt\right)}^{\left[\frac{4}{f\left(\alpha \right)}\right]-2}\partial^{i}\frac{\partial^{l}\partial_{l}\sqrt{\rho }}{\sqrt{\rho }}$$

a quantity which can be viewed as a measure of the multifractality of the motion curves of the SMA dynamics.

It is noted that that for various constraints, in particular, for a scalar potential *U*, Equation (5) becomes:

$$\frac{d{\hat{V}}^{i}}{dt}=\partial_{t}{\hat{V}}^{i}+{\hat{V}}^{l}\partial_{l}{\hat{V}}^{i}+\frac{1}{4}{\left(dt\right)}^{\left[\frac{2}{f\left(\alpha \right)}\right]-1}D^{lk}\partial_{l}\partial_{k}{\hat{V}}^{i}=\partial^{i}\left(U\right)$$

For multifractalization by means of Markov-type stochastic processes, the above equation takes the form:

$$\frac{\hat{d}{\hat{V}}^{i}}{dt}=\partial_{t}{\hat{V}}^{i}+{\hat{V}}^{l}\partial_{l}{\hat{V}}^{i}-i\lambda {\left(dt\right)}^{\left[\frac{2}{f\left(\alpha \right)}\right]-1}\partial_{l}\partial^{l}{\hat{V}}^{i}=\partial^{i}\left(U\right)$$

Considering the above, the multifractal hydrodynamic equations become:

(18) $$\partial_{t}V_{D}^{i}+V_{D}^{l}\partial_{l}V_{D}^{i}=-\partial^{i}\left(Q+U\right)$$

(19) $$\partial_{t}\rho +\partial_{l}\left(\rho V_{D}^{l}\right)=0$$

Several consequences which result from Equations (18) and (19) are given in [38,42].

If employing the tensor,

(20) $${\hat{\tau }}^{il}=2\lambda^{2}{\left(dt\right)}^{\left[\frac{4}{f\left(\alpha \right)}\right]-2}\rho \partial^{i}\partial^{l}\ln\rho$$

and Equation (17) becomes the multifractal equilibrium equation:

(21) $$\rho \partial^{i}Q=\partial_{l}{\hat{\tau }}^{il}$$

Another form of Equation (20) can then be:

(22) $${\hat{\tau }}^{il}=\eta \left(\partial_{l}V_{F}^{i}+\partial_{i}V_{F}^{l}\right)$$

with:

(23) $$\eta =\lambda {\left(dt\right)}^{\left[\frac{2}{f\left(\alpha \right)}\right]-1}\rho$$

This represents a multifractal linear constitutive equation for a multifractal “viscous fluid”, where the coefficient *η* is the multifractal dynamic viscosity of the multifractal fluid. The presence of the tensor can be attributed to the martensitic–austenitic transition of the SMA.

### 2.2. Mechanical Hysteresis-Type Behaviors “Mimed” through a Multifractal Tunnel Effect

**Figure 1:**
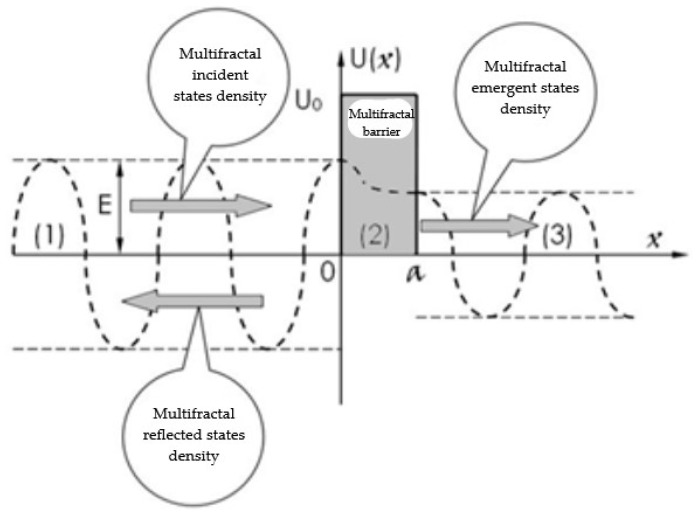
Multifractal barrier—SMA system describing the tunnel effect.

Some assumptions can be made regarding SMA dynamics:

- Any SMA can be viewed as a mathematical object of multifractal type;
- Any SMA dynamics can be described by means of multifractal hydrodynamic equations;
- The SMA system functions as a multifractal tunnel effect defined by the scalar potential (see Figure 1).

(24) $$U\left(x\right)=\left\{\begin{array}{l} 0\;-\infty <x<0\\ U_{0}\;0\leq x\leq a\\ 0\;a<x<+\infty \end{array}\right.$$

In Equation (24), *U*_0_ is the barrier height and *a* is its width (i.e., the characteristics of the SMA system).

- SMA dynamics can be defined by means of the multifractal energy conservation law in the shape:
  (25) Q+U=E
  or explicitly:
  (26) $$2\lambda^{2}{\left(dt\right)}^{\left[\frac{4}{f\left(\alpha \right)}\right]-2}\frac{\partial^{l}\partial_{l}\sqrt{\rho }}{\sqrt{\rho }}+U=E$$

The quantities present in Equation (26) have their meanings given in [38,42].

Taking into account the one-dimensional case Equation (26), by substitution,

(27) $$\sqrt{\rho }=\theta \left(x\right)$$

becomes:

(28) $$\partial_{xx}\theta \left(x\right)+\frac{1}{2\lambda^{2}{\left(dt\right)}^{\left[\frac{4}{f\left(\alpha \right)}\right]-2}}\left(E-U\right)\theta \left(x\right)=0$$

As portrayed in Figure 1, it is possible to define three distinct areas (zones), described as:

- Zone (1), named the multifractal incidence zone;
- Zone (2), named the multifractal barrier;
- Zone (3), named the multifractal emergence zone.

In such a context, if $\theta_{1}$, $\theta_{2}$ and $\theta_{3}$ are the multifractal functions corresponding to the three previously mentioned zones, the following equations emerge:

(29a) $$\frac{d^{2}\theta_{1}}{dx^{2}}+k^{2}\theta_{1}=0,\;-\infty <x<0$$

(29b) $$\frac{d^{2}\theta_{2}}{dx^{2}}-q^{2}\theta_{2}=0,\;0\leq x\leq a$$

(29c) $$\frac{d^{2}\theta_{3}}{dx^{2}}+k^{2}\theta_{3}=0,a<x<+\infty$$

where:

(30) $$k^{2}=\frac{E}{2\lambda^{2}{\left(dt\right)}^{\left(4/f\left(\alpha \right)\right)-2}},q^{2}=\frac{U_{0}-E}{2\lambda^{2}{\left(dt\right)}^{\left(4/f\left(\alpha \right)\right)-2}}$$

By means of integration, the solutions of the above equations are of the form:

(31a) $$\theta_{1}\left(x\right)=A_{1}e^{ikx}+B_{1}e^{-ikx},\;\;-\infty <x<0$$

(31b) $$\theta_{2}\left(x\right)=A_{2}e^{qx}+B_{2}e^{-qx},\;\;0\leq x\leq a$$

(31c) $$\theta_{3}\left(x\right)=A_{3}e^{ikx},\;a<x<+\infty$$

where *A*_1_, *B*_1_, *A*_2_, *B*_2_ and *A*_3_ are constants. The meanings of these solutions are given in [43].

Because the multifractal current density in the one-dimensional case is given by [38,41,42,43]:

(32) $$J_{x}=i\lambda {\left(dt\right)}^{\left(2/f\left(\alpha \right)\right)-1}\left(\theta \frac{d\bar{\theta }}{dx}-\bar{\theta }\frac{d\theta }{dx}\right)$$

then, in accordance with [43], it is possible to distinguish:

- For Zone (1) (incident states):

(33) $$J_{i}=2\lambda {\left(dt\right)}^{\left(\frac{2}{f\left(\alpha \right)}\right)-1}k{\left|A_{1}\right|}^{2}$$

- For Zone (3) (emergent states):

(34) $$J_{e}=2\lambda {\left(dt\right)}^{\left(2/f\left(\alpha \right)\right)-1}k{\left|A_{3}\right|}^{2}$$

- For Zone (1) (reflected states):

(35) $$J_{r}=-2\lambda {\left(dt\right)}^{\left(2/f\left(\alpha \right)\right)-1}{\left|B_{1}\right|}^{2}$$

The previously presented results allow the univocal characterization of the multifractal tunnel effect by means of the multifractal transparency:

(36) $$T=\frac{J_{e}}{J_{i}}={\left|\frac{A_{3}}{A_{1}}\right|}^{2}$$

and the multifractal reflectance:

(37) $$R=\frac{J_{r}}{J_{i}}={\left|\frac{B_{1}}{A_{1}}\right|}^{2}$$

With the coupling conditions (in *x* = 0 and *x* = *a*), for the functions *θ*_i_ and their derivates, i.e.,

(38a) $$\theta_{1}\left(0\right)=\theta_{2}\left(0\right)$$

(38b) $$\frac{d\theta_{1}}{dx}\left(0\right)=\frac{d\theta_{2}}{dx}\left(0\right)$$

(38c) $$\theta_{2}\left(a\right)=\theta_{3}\left(a\right)$$

(38d) $$\frac{d\theta_{2}}{dx}\left(a\right)=\frac{d\theta_{3}}{dx}\left(a\right)$$

the multifractal algebraic system results:

(39a) $$A_{1}+B_{1}=A_{2}+B_{2}$$

(39b) $$ik\left(A_{1}-B_{1}\right)=q\left(A_{2}-B_{2}\right)$$

(39c) $$e^{qa}A_{2}+e^{-qa}B_{2}=e^{iqa}A_{3}$$

(39d) $$q\left(e^{qa}A_{2}-e^{-qa}B_{2}\right)=ike^{iqa}A_{3}$$

As a consequence, according to [43], the multifractal transparency becomes:

(40) $$T=\frac{4q^{2}k^{2}}{4q^{2}k^{2}+{\left(q^{2}+k^{2}\right)}^{2}sh^{2}\left(qa\right)}$$

and the multifractal reflectance takes the form:

(41) $$R=\frac{{\left(k^{2}+q^{2}\right)}^{2}}{{\left(q^{2}-k^{2}\right)}^{2}+4q^{2}k^{2}\cdot cth^{2}\left(qa\right)}$$

Furthermore, by referring to Equation (30), it is possible to obtain:

(42) $$R=\frac{U_{0}^{2}sh^{2}\left\{{\left[\frac{\left(U_{0}-E\right)}{2\lambda^{2}{\left(dt\right)}^{\left(4/f\left(\alpha \right)\right)-2}}\right]}^{1/2}a\right\}}{U_{0}^{2}sh^{2}\left\{{\left[\frac{\left(U_{0}-E\right)}{2\lambda^{2}{\left(dt\right)}^{\left(4/f\left(\alpha \right)\right)-2}}\right]}^{1/2}a\right\}+4E\left(U_{0}-E\right)}$$

(43) $$T=\frac{4E\left(U_{0}-E\right)}{U_{0}^{2}sh^{2}\left\{{\left[\frac{\left(U_{0}-E\right)}{2\lambda^{2}{\left(dt\right)}^{\left(4/f\left(\alpha \right)\right)-2}}\right]}^{1/2}a\right\}+4E\left(U_{0}-E\right)}$$

It is also possible to make use of the dimensionless coordinate system:

(44a) $$X=ka={\left[\frac{E}{2\lambda^{2}{\left(dt\right)}^{\left(\frac{4}{f\left(\alpha \right)}\right)-2}}\right]}^{\frac{1}{2}}a$$

(44b) $$Y=qa={\left[\frac{\left(U_{0}-E\right)}{2\lambda^{2}{\left(dt\right)}^{\left(4/f\left(\alpha \right)\right)-2}}\right]}^{\frac{1}{2}}a$$

a situation in which Equations (42) and (43) become:

(45) $$R=\frac{{\left(X^{2}+Y^{2}\right)}^{2}}{{\left(Y^{2}-X^{2}\right)}^{2}+4X^{2}Y^{2}cth^{2}\left(Y\right)}$$

(46) $$T=\frac{4X^{2}Y^{2}}{4X^{2}Y^{2}+{\left(X^{2}+Y^{2}\right)}^{2}sh^{2}\left(Y\right)}$$

**Figure 2:**
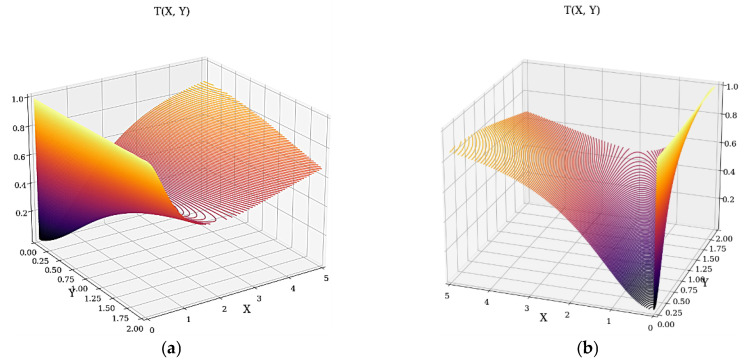
(**a**,**b**) The 3D dependencies *T* = *T* (X, Y) from two perspectives.

In what follows, the 3D dependence of *T* on X and Y is shown in Figure 2a,b.

**Figure 3:**
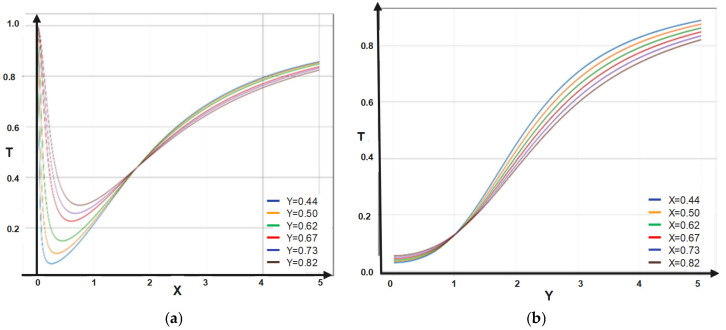
(**a**,**b**) The 2D dependencies of *T* = *T* (X, Y): (**a**) *T* = *T* (X, Y = constant); (**b**) *T* = *T* (X = constant, Y).

The 2D dependencies of *T* on X and Y are depicted in Figure 3a,b.

**Figure 4:**
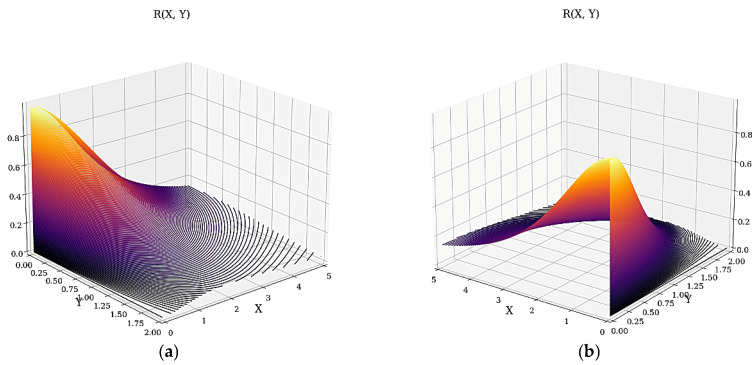
(**a**,**b**) The 3D dependencies *R* = *R* (X, Y) from two perspectives.

In Figure 4a,b, the 3D dependencies of *R* on X and Y are given.

**Figure 5:**
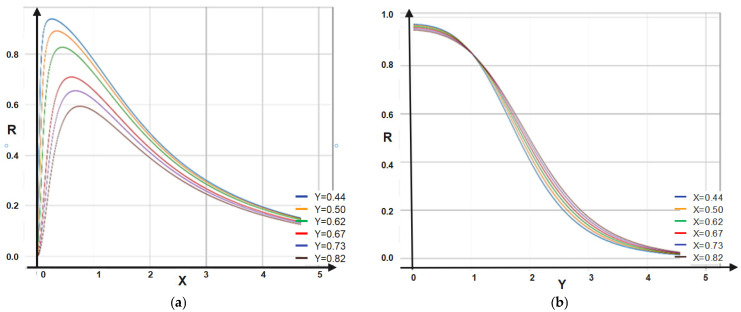
(**a**,**b**) The 2D dependencies of *R* = *R* (X, Y): (**a**) *R* = *R* (X, Y = constant); (**b**) *R* = *R* (X = constant, Y).

In Figure 5a,b the 2D dependencies of *R* on X and Y are given.

The dependence of the multifractal transparency on X entails minimal and asymptotic increases in T. On Y, it entails only asymptotic increases in T. The dependence of the multifractal reflectance on X entails maximal and asymptotic decreases in R. On Y, it entails only asymptotic decreases in R.

**Figure 6:**
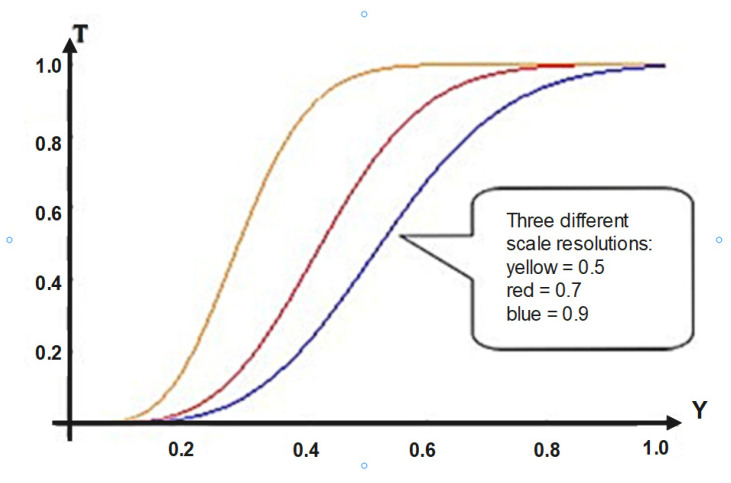
Detail of the variation in the transparency coefficient at three different scale resolutions, for a variation in Y in the interval 0 ÷ 1.

In the context of the statements made above, in Figure 6, details on the dependence *T* = *T*(Y) in the interval 0 ÷ 1 are given so. It is possible to “mime” mechanical hysteresis-type behaviors for SMA systems if we match in correspondence the dependence *T* = *T*(Y) with the dependence force *(F)*—a function of displacement (Δ*l*), i.e., *F* = *F*(Δl). Such a correspondence is obviously possible given the significance of the magnitudes involved in both *T* and *Y*.

## 3. Experimental Design of SMAs of Cu–Al–Zn Alloy

For the purpose of studying the mechanical properties, nine samples in total were prepared, with different percentages of the metals found in the Cu–Al–Zn SMA. When establishing the chemical composition of the alloy, a series of factors must be taken into account (see [44]). The samples were thermally cured at the temperature of 800 °C for a time interval of one hour, and afterwards, they were quickly quenched in water.

The variation in the weight percentages of metal from the composition of the alloy was an important criterion for the following research stages (see Table 1).

For this study, a Universal Testing Machine 60 kN, controlled with WinWDW software (http://cloudserver015871.home.pl/images/img/wdw.pdf, accessed on 3 February 2023)—which collects real-time data—was used. The procedure of data collection consisted in continuous readings of the force, for each sample, up until a relative elongation of 1.5% and of 3%. After each elongation, the force ceased its action and the sample returned to its original form.

In order to avoid any possible damage to the samples due to the clamps installed on the testing machine, end support plates were affixed to the samples.

The samples were tested in tension until the relative elongation registered by the press reached approximately ε = 2%. After reaching the target value, the force slowly decreased until zero. Both the loading and unloading speed were set at v = 0.2 kN/s. The loading–unloading process was repeated five times for each sample, in order to eliminate any possible errors [44].

**Figure 7:**
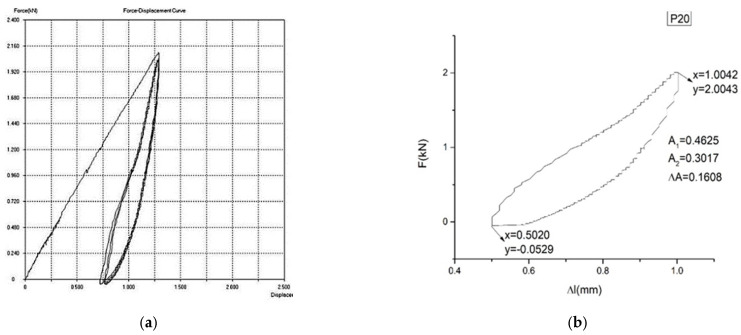
Force–displacement diagram for sample P20: (**a**) F = f(Δl) for 5 cycles of loading–unloading. (**b**) F = f(Δl) for the last cycle of loading–unloading. The yield of sample P20 is η = 34.76%.

**Figure 8:**
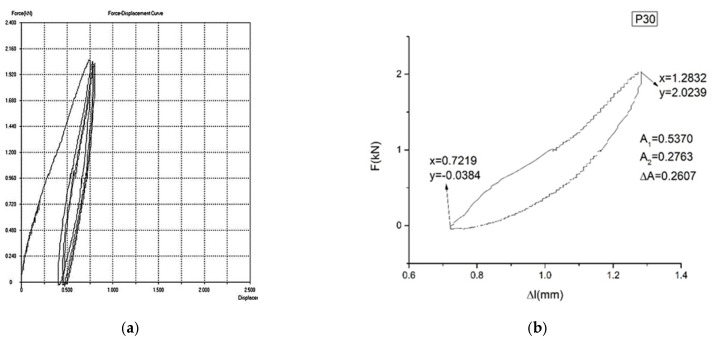
Force–displacement diagram for sample P30: (**a**) F = f(Δl) for 5 cycles of loading–unloading. (**b**) F = f(Δl) for the last cycle of loading–unloading. The yield of sample P30 is η = 48.54%.

**Figure 9:**
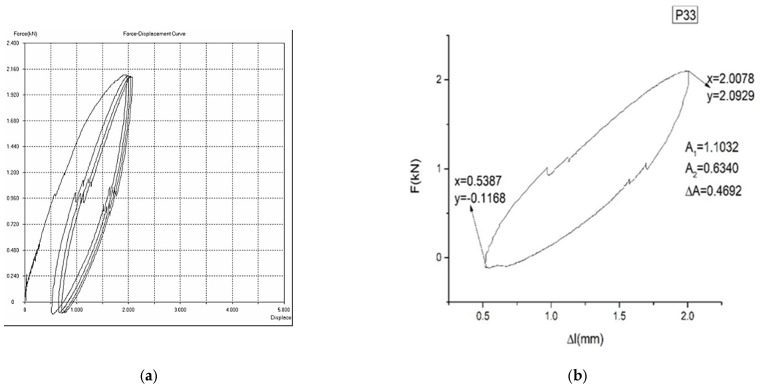
Force–displacement diagram for sample P33: (**a**) F = f(Δl) for 5 cycles of loading–unloading. (**b**) F = f(Δl) for the last cycle of loading–unloading. The yield of sample P33 is η = 42.53%.

In Figure 7a,b, Figure 8a,b and Figure 9a,b, the force–displacement diagrams are presented for three representative samples.

ΔA is proportional to the dissipated energy in a loading–unloading cycle. $A_{1}$ is proportional to the loading energy. $A_{2}$ is proportional to the unloading energy. The smaller ΔA is, the better the yield ($\eta =\frac{\left(A_{1}-A_{2}\right)}{A_{1}}$) of the material transformation is (and, therefore, a better mechanical memory can be observed). From the representative samples, P30 has the best yield.

It is mentioned here that the curves presented in Figure 7, Figure 8 and Figure 9 are pseudotwining elastic curves [45]. As a consequence, the reversible deformation highlighted during the five loading–unloading cycles does not exhibit a plateau at constant force, typical for super-elasticity in an austenitic state, as is observed in typical super-elastic alloys (e.g., Ni–Ti SMAs). For example, for sample P33, the maximum force for which the loading–unloading cycles were carried out was 200 kN, corresponding to a displacement of 2%.

**Figure 10:**
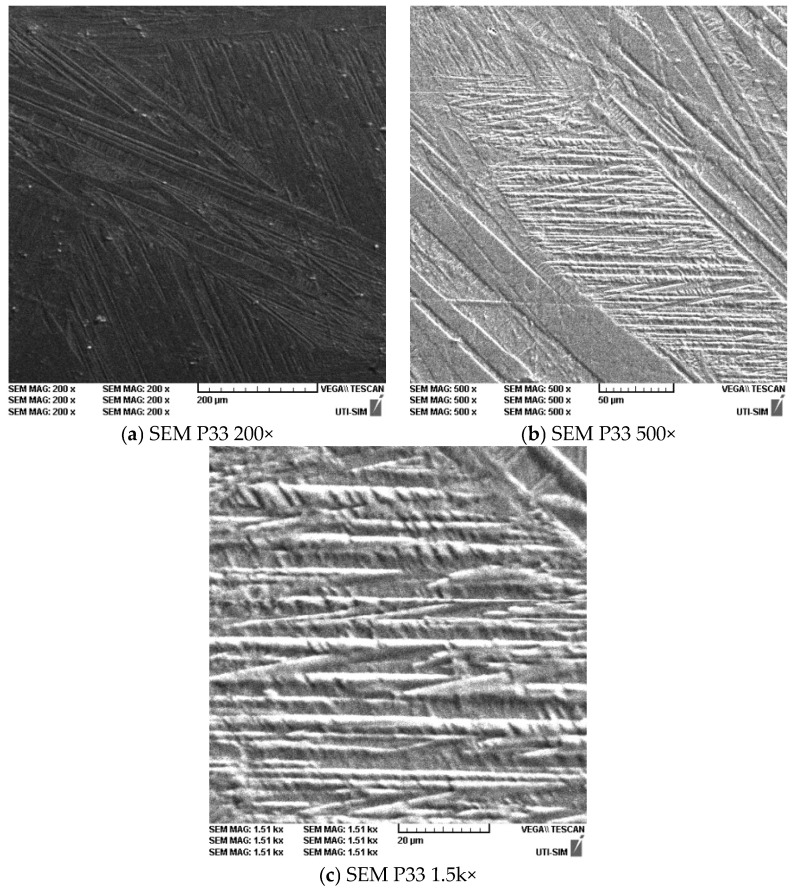
SEM results for sample P33 at various zooming scales: (**a**)-200×; (**b**)-500×; (**c**)-1500×.

The average grain size was 50 µm. Microstructure details are presented in Figure 10a–c for the sample P33. The highlighting of the microstructure was realized using SEM, at ambient temperature, for different zooming scales. It is possible to observe the martensitic varieties, oriented through self-accommodation, following the sample treatment. The martensitic varieties are presented in various morphological types (plates, zig-zag, diamond, pike nose), together with grain limits and three-grain intersection.

## 4. Validation of the Model

It is well-known that the properties of SMAs can be highlighted through various theoretical models that link with experimental data (both analytical and computational models—for details, see [46]). Taking into account the fact that SMAs, both structurally and functionally “mime” fractal/multifractal-type behaviors (see fractal structures in SMAs [47] and patterns and chaotic behaviors in [48]), in Section 2, a new model to analyze the dynamics of SMAs was proposed. The starting point is the hypothesis that both structurally and functionally, the SMA can be assimilated with a mathematical object of fractal/multifractal type (i.e., in the most general case, with a complex system). Then, the dynamics of the structural units of such a system can be described through continuous and non-differentiable curves (fractal/multifractal curves). The model which allows such an approach is the one given in [37,38,39]. In such a context, the fractalization/multifractalization processes are realized through stochasticization (in the proposed model, through Markov/non-Markov stochastic processes, in contrast with other stochastic processes from [47,48]). This allows the “miming” of SMA dynamics based on fractal/multifractal fluid-type behaviors (for details, see [38,39]). Once accepting the functionality of such a model, the measuring of the fractal/multifractal degree can be put in correspondence with the scalar fractal/multifractal potential, according to [37,38,39]. Thus, the group invariance of the SL(2R) type of said potential allows for the development of Riemannian manifolds, which easily allow the estimation of areas between the fractality/multifractality curves between two fixed points of a manifold. In such a context, through harmonic mappings between the usual space (the Euclidean space) and the Riemann space, it is possible to estimate the said areas (i.e., the ones in the Euclidean space). All this mathematical methodology is extensively presented in [37,38,39]. Taking into account the mathematical difficulty of linking theoretical models with experimental data (for example, regarding the computation of areas), in a future paper, it is proposed to tackle such a subject.

Now, as it can be seen from Figure 6, the theoretical model can provide a way to “mime” the experimental curves presented in Figure 7, Figure 8 and Figure 9. In the current case, in the way the physical quantities T and Y are defined, a direct correspondence with the measurable quantities, i.e., the force and displacement, can be established (the force can be correlated with T and the displacement can be correlated with Y), all these being given in dimensionless coordinates. In such a context, the chemical composition can be correlated with the shapes of the T(Y) curves (controlled through the multifractality degree), while the areas delimited by the T(Y) curves can be correlated with the multifractal specific potential, as a measure of the mechanical memory degree.

## 5. Conclusions

The main conclusions of the present paper are the following:

○ By assimilating SMAs with mathematical multifractal-type objects, a theoretical model, using the Scale Relativity Theory, was developed for the purpose of explaining the behavior of such materials;
○ Considering that the dynamics of the entities belonging to any SMA are described through continuous and non-differentiable curves (multifractal curves), the motion equations (geodesics on multifractal manifolds) were obtained in the multifractal hydrodynamic model;
○ The properties of any SMA were put in correspondence with the properties of a multifractal fluid: complex viscous-type coefficient in correlation with the scale resolution (which can “mime” the shape memory), the multifractal reversibility in correlation with the martensite–austenite transformation, and the existence of a multifractal tensor, which is in correlation with material constitutive laws (in particular, the force–displacement curve);
○ As a final remark, the chemical composition of the SMA can be linked to the fractality degree, while the yield can be linked to the scale resolution, as is shown in Figure 6;
○ The loading–unloading graphs of the alloys show a pronounced hysteresis. This is due to the fact that the inverse transformation (martensite–austenite) does not occur at the same stress levels during unloading, compared to the direct transformation during loading. This indicates that a supplementary force is needed due to stored elastic deformation energy;
○ When in the loading phase, the alloy undergoes a transformation from the austenitic phase to the martensitic phase;
○ When the force reaches a maximum, the original austenitic phase is transformed into a martensitic one. An elastic loading of the martensite might occur (accompanied by a percentage of non-transformable, residual austenite) which, in practice, can lead to a conventional plastic deformation above this loading leve;
○ If the martensitic material is too tensed, an irreversible plastic deformation may occur;
○ When the force decreases, the alloy reverses to its initial phase, this aspect being possible due to the retransformations stress (which means that hysteresis exists);
○ Finally, for a sufficiently low load, the alloy completely reverts to its austenitic phase.

## Figures and Tables

**Table 1:** The weight percentages of metal from the composition of the samples made of Cu–Al–Zn alloy.

Sample No.	Cu Percentage(%)	Zn Percentage(%)	Al Percentage(%)	Trace Material Percentage(%)
P10	71.01	22.52	6.41	0.039
P12	78.59	15.21	6.13	0.042
P17	74.50	18.40	7.06	0.035
P19	75.08	18.05	6.78	0.079
P20	74.06	20.86	6.69	0.097
P30	73.63	19.40	6.90	0.042
P33	70.29	25.17	4.40	0.054
P34	74.06	21.57	4.31	0.034
P36	72.60	21.21	6.12	0.11

## Data Availability

Not applicable.
